# “Examiners” Wanted

**Published:** 1873-02-01

**Authors:** 


"Examiners" Wanted.—If any of our readers have a copy of The Medical Examiner for July 1st, 1872, which they do not care to keep, and they will mail it to the senior editor, he will pay them double the subscription price. We are very desirous of obtaining three or four copies of that number.

